# Effects of the ZrO_2_ Crystalline Phase and Morphology on the Thermocatalytic Decomposition of Dimethyl Methylphosphonate

**DOI:** 10.3390/nano14070611

**Published:** 2024-03-30

**Authors:** Xuwei Wang, Peng Sun, Ziwang Zhao, Yimeng Liu, Shuyuan Zhou, Piaoping Yang, Yanchun Dong

**Affiliations:** 1 Key Laboratory of Superlight Materials and Surface Technology, Ministry of Education, College of Material Sciences and Chemical Engineering, Harbin Engineering University, Harbin 150001, China; wangxw5710@hrbeu.edu.cn (X.W.); pengs@hrbeu.edu.cn (P.S.); 2 State Key Laboratory of NBC Protection for Civilian, Beijing 102205, China; zhaoziwang1123@163.com (Z.Z.); 202311087233@mail.scut.edu.cn (Y.L.)

**Keywords:** dimethyl methylphosphonate (DMMP), thermocatalytic decomposition, ZrO_2_ catalyst, morphology, crystalline phase

## Abstract

Thermocatalytic decomposition is an efficient purification technology that is potentially applicable to degrading chemical warfare agents and industrial toxic gases. In particular, ZrO_2_ has attracted attention as a catalyst for the thermocatalytic decomposition of dimethyl methylphosphonate (DMMP), which is a simulant of the nerve gas sarin. However, the influence of the crystal phase and morphology on the catalytic performance of ZrO_2_ requires further exploration. In this study, monoclinic- and tetragonal-phase ZrO_2_ (m- and t-ZrO_2_, respectively) with nanoparticle, flower-like shape and hollow microsphere morphologies were prepared via hydrothermal and solvothermal methods, and their thermocatalytic decomposition of DMMP was systematically investigated. For a given morphology, m-ZrO_2_ performed better than t-ZrO_2_. For a given crystalline phase, the morphology of hollow microspheres resulted in the longest protection time. The exhaust gases generated by the thermocatalytic decomposition of DMMP mainly comprised H_2_, CO_2_, H_2_O and CH_3_OH, and the by-products were phosphorus oxide species. Thus, the deactivation of ZrO_2_ was attributed to the deposition of these phosphorous oxide species on the catalyst surface. These results are expected to help guide the development of catalysts for the safe disposal of chemical warfare agents.

## 1. Introduction

Sarin (i.e., isopropyl methylphosphonofluoridate) is a nerve agent in vapour form with military applications [1] that enters the human body through respiration or via the skin and eyes to bind to acetylcholinesterase within the body. Just a small amount of short-term inhalation can damage the nervous system, and long-term excessive inhalation can have lethal effects [2]. The primary method for removing sarin is chromium-free impregnated carbon adsorption [3], but this technology only transfers sarin into the pores of the adsorption material, which can easily result in secondary pollution. Therefore, the adsorption material requires additional treatment to fully eliminate sarin. This had led to extensive efforts both domestically and internationally to develop innovative purification technologies against nerve agents. Owing to the extreme toxicity of sarin, dimethyl methylphosphonate (DMMP) has usually been used in experiments as a simulant because it is less toxic while having a similar structure.

Thermocatalytic decomposition involves using catalysts to reduce the activation energy and carrying out flameless combustion at lower ignition temperatures. The toxic molecules in the contaminated air adsorb onto the active sites of the catalysts, which results in a series of chemical decomposition effects that generate small molecular compounds such as CO_2_ and H_2_O. Thermocatalytic decomposition is widely applicable and efficient, and this purification technology is expected to replace conventional adsorption technologies [4,5,6,7,8,9,10]. Most studies on the thermocatalytic decomposition of DMMP have focused on using multivalent metal oxides [11,12,13,14]. Walenta et al. [14] investigated the DMMP decomposition performance of iron oxide and found that DMMP dissociates to form methoxy and methylphosphonic acid methyl ester (CH_3_O)P(O)_2_(CH_3_) at room temperature. At elevated temperatures, DMMP decomposes to form dimethyl ether, which reacts with lattice oxygen to generate reaction products such as H_2_ and CO_2_, and phosphorus oxide species accumulate as by-products on the catalyst surface. Gao et al. [12] prepared a loaded Cu–Ce catalyst using the equal-volume impregnation method. Their results indicated a strong interaction between Cu and Ce on CuO-5%CeO_2_/γ-Al_2_O_3_ and the promotion of surface-adsorbed oxygen, which improved the thermocatalytic decomposition of DMMP.

Zirconia (ZrO_2_) is a high-temperature-resistant metal oxide with acidic, alkaline, oxidative and reductive properties that has attracted tremendous attention in the fields of alkane conversion and catalytic cracking of gasoline [15,16,17]. Several studies have considered applying zirconium oxides as catalysts for the adsorption of DMMP [18,19]. Long et al. [19] synthesised zirconium oxide aerogels (ZrO_x_H_y_) using propylene oxide and aqueous ZrCl_4_ as raw materials and used in situ infrared spectroscopy to investigate their performance as an active adsorbent for DMMP. Their results indicated that DMMP rapidly decomposed when it reacted with hydroxyl-rich aerogels, which generated surface-bound Zr–OCH_3_ and bridged O–P–O species. Meanwhile, amorphous zirconium hydroxide has emerged as a material for degrading nerve agents and toxic industrial chemicals [20,21]. Jeon et al. [20] prepared nanoscale amorphous zirconium hydroxide films on a metal substrate by cathodic electrodeposition of ZrO_2_ in an aqueous solution and used in situ atomic absorption spectroscopy to investigate the atmospheric pressure adsorption and decomposition kinetics of DMMP. Based on the characteristics of the vibration spectrum, they identified reaction products, including bridged, chelated and monodentate methyl phosphonate along with bridged monodentate methoxy.

Although some progress has been made in research on using ZrO_2_ as a catalyst and adsorbent of DMMP at room temperature, studies on its application to the thermocatalytic decomposition of DMMP are still lacking. In particular, the influence of the ZrO_2_ morphology and crystalline phase should be considered. In this study, six types of ZrO_2_ were prepared with different morphologies (i.e., nanoparticle, flower-like shape and hollow microsphere) and crystalline phases (i.e., monoclinic and tetragonal), and the effects on the thermocatalytic decomposition of DMMP were evaluated. The experimental results were used to analyse the reaction mechanism and reasons for catalytic deactivation.

## 2. Materials and Methods

### 2.1. Preparation of Catalysts

The hydrothermal and solvothermal methods were used to prepare monoclinic- and tetragonal-phase ZrO_2_ (m-ZrO_2_ and t-ZrO_2_, respectively) with different morphologies.

#### 2.1.1. Nanoparticles

To prepare m- and t-ZrO_2_ nanoparticles, a modified version of Li et al.’s [22] method was used. For the m-ZrO_2_ nanoparticles, 7.728 g of ZrOCl_2_·8H_2_O was dissolved in 60 mL of CH_3_OH solution and stirred. Then, 14.4 g of CO(NH_2_)_2_ was added and stirred until a transparent solution was obtained. This solution was transferred to a 100 mL Teflon-lined autoclave, which was placed in an oven at 160 °C for 20 h. The reaction product was washed with water and ethanol and centrifuged until no white precipitate formed when AgNO_3_ solution was added. The centrifuged product was then dried at 60 °C for 10 h and was calcined in air at a heating rate of 10 °C/min to 400 °C, where it was kept for 4 h to obtain the m-ZrO_2_ nanoparticles. For the t-ZrO_2_ nanoparticles, the same procedure as for the m-ZrO_2_ nanoparticles was used except that calcining took place in a N_2_ atmosphere rather than in air.

#### 2.1.2. Flower-like Shape

To prepare m- and t-ZrO_2_ with a flower-like shape, a modified version of Shu et al.’s [23] method was used. For m-ZrO_2_ with a flower-like shape, 1.755 g of Zr(SO_4_)_2_ was dissolved in 60 mL of deionised water and stirred. Then, 0.3 g of CH_3_COONa was added and stirred until a transparent solution was obtained. The solution was transferred to a 100 mL Teflon-lined autoclave, which was placed in an oven at 200 °C for 6 h. The reaction product was washed with water and ethanol, centrifuged three times, dried at 60 °C for 8 h and calcined in air at a heating rate of 5 °C/min to 800 °C, where it was kept for 4 h to obtain m-ZrO_2_ with a flower-like shape. For t-ZrO_2_ with a flower-like shape, the same procedure was used except that calcining took place in air at a heating rate of 10 °C/min to 600 °C, which was then kept for 4 h.

#### 2.1.3. Hollow Microspheres

To prepare m-ZrO_2_ hollow microspheres [24], 1.5 g of ZrOCl_2_·8H_2_O was dissolved in 60 mL of anhydrous ethanol and stirred. Then, 0.9 g of CO(NH_2_)_2_ was added and mixed. Next, 15 mL of 36.5% HCl was added dropwise to form a transparent mixed solution. The solution was transferred to a 100 mL Teflon-lined autoclave, which was placed in an oven at 160 °C for 24 h. The reaction product was washed with water and ethanol, and centrifuged until no white precipitate formed when AgNO_3_ solution was added. The centrifuged product was dried at 60 °C for 6 h and calcined in air at a heating rate of 10 °C/min to 450 °C, where it was kept for 4 h to obtain m-ZrO_2_ hollow microspheres.

To prepare t-ZrO_2_ hollow microspheres [25], 0.966 g of ZrOCl_2_·8H_2_O and 0.099 g of Y(NO_3_)_3_·6H_2_O were dissolved in 60 mL of a mixed solution of n-butanol and acetylacetone (1:1 volume ratio) and stirred until a transparent solution was obtained. The solution was transferred to a 100 mL Teflon-lined autoclave, which was placed in an oven at 65 °C for 4 h and then reacted at 200 °C for 12 h. The reaction product was washed with water and centrifuged until no white precipitate formed when the AgNO_3_ solution was added. The centrifuged product was then vacuum dried at 80 °C for 5 h to obtain t-ZrO_2_ hollow microspheres.

### 2.2. Characterization

The following methods were used to characterize the prepared catalysts. To analyse the crystalline structure, X-ray diffraction (XRD) (Rigaku SmartLab SE, Saitama, Japan) was performed at a scanning speed of 2°/min and scanning range of 5–90°. To observe the particle size and morphology, scanning electron microscopy (SEM) (TESCAN MIRA LM, Brno, Czechia) with a gold sputter coating and high-resolution transmission electron microscopy (TEM) (JEOL JEM F200, Tokyo, Japan) were used. Brunauer–Emmett–Teller (BET) analysis was performed by using an automatic surface area analyser (Quantachrome Autosorb IQ, Boynton Beach, FL, USA) to obtain the specific surface area. Before testing, all samples were degassed at 300 °C for 6 h. Tests were then performed in a liquid nitrogen environment, and the corresponding specific surface area was calculated by using the BET method. The redox properties were evaluated by using the hydrogen temperature-programmed reduction (H_2_-TPR) technique with a chemical adsorption instrument (Micromeritics AutoChem II 2920, Norcross, GA, USA). In each test, 50 mg of a sample was dried at 300 °C under a He flow at a flow rate of 50 mL/min for 2 h. The sample was then cooled to 50 °C and treated with a 10% H_2_/Ar gas mixture at a flow rate of 50 mL/min for 0.5 h. When the baseline became stable, the sample was desorbed in a 10% H_2_/Ar flow at a heating rate of 10 °C/min up to 900 °C. The reduction gas was detected by using a thermal conductivity detector. X-ray photoelectron spectroscopy (XPS) (Thermo Scientific K-Alpha, Waltham, MA, USA) was used to study the valence states of catalyst elements. The binding energy was calibrated using the C1s peak of 284.8 eV, and the XPS data was fitted using advanced software.

### 2.3. Performance Evaluation

**Figure 1:**
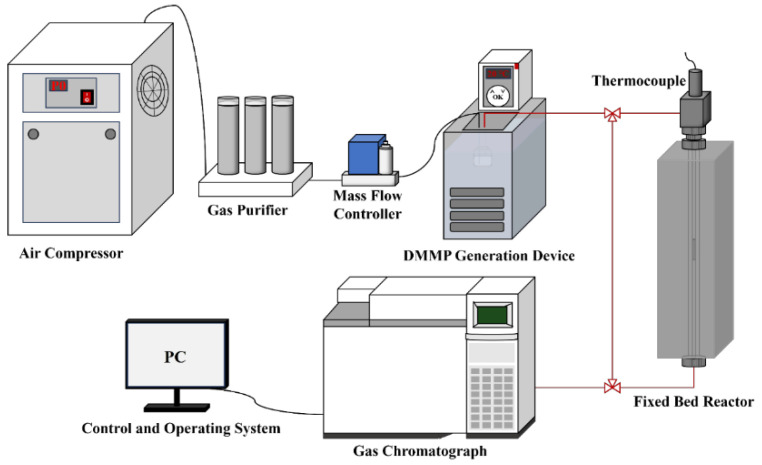
Schematic of the custom-built apparatus for evaluation of the thermocatalytic decomposition performance.

Figure 1 shows the custom-built apparatus used to evaluate the thermocatalytic decomposition performance of the prepared catalysts. The apparatus comprised a DMMP generation device, fixed-bed reactor for the catalytic reaction and device for exhaust gas analysis. DMMP vapour was generated by the bubbling method, which introduced 50 mL/min of purified air into a DMMP generation bottle. The bottle was placed in a water bath at a constant temperature of 30 °C, and the DMMP concentration was controlled at 5.3 g/m^3^. A quartz tube with an inner diameter of 4 mm was placed in a vertical tubular resistance furnace with a programmable temperature control to act as the fixed-bed reactor. Each catalyst sample was pressed, crushed and sieved (20–40 mesh), after which it was used to fill the quartz tube. Both ends of the catalyst sample were fixed with quartz cotton, and the temperature of the fixed-bed reactor was set to 400 °C. The filling height was 2.4 cm, and the gas hourly space velocity was 9952 h^−1^. The loading amounts of m-ZrO_2_ nanoparticles, t-ZrO_2_ nanoparticles, m-ZrO_2_ flower-like shapes, t-ZrO_2_ flower-like shapes, m-ZrO_2_ hollow microspheres, and t-ZrO_2_ hollow microspheres were 0.4105, 0.4005, 0.4392, 0.4265, 0.3753, and 0.3762 g, respectively. The exhaust gas analysis device comprised a gas chromatograph (Agilent 6890N, Santa Clara, CA, USA) equipped with a flame ionisation detector and a DB-1701 column connected to a computer control terminal so that the changes in the DMMP concentration before and after the thermocatalytic decomposition could be monitored online. The generation bottles, fixed-bed reactor, and gas chromatograph were connected by pipelines kept at a constant temperature of 80 °C to prevent the DMMP vapour from condensing. To verify the thermal stability of DMMP at 400 °C, a blank experiment was conducted by introducing DMMP into an empty quartz tube without any catalyst. To minimise potential errors, complete DMMP conversion was defined as a conversion rate of ≥99%, and the protection time was defined as the amount time for complete DMMP conversion. The protection time is widely used to evaluate the thermocatalytic decomposition performance of a catalyst [26]. The conversion rate (${CVR}_{DMMP}$) is expressed as follows:

(1) $${CVR}_{DMMP}=(1-\frac{C_{out}}{C_{in}})\times 100\%$$

where *C_out_* is the DMMP concentration in the exhaust gases after thermocatalytic decomposition, and *C_in_* is the initial DMMP concentration generated by the bubbling.

### 2.4. Qualitative Analysis of the Exhaust Gases

After the thermocatalytic decomposition, the gaseous products were qualitatively analysed by using a microreactor (CATLAB) equipped with a mass spectrometer (HPR-20, Hiden Ltd., Warrington, UK). First, pure He was vented at a rate of 80 mL/min into the microreactor, which was loaded with 100 mg of a catalyst at room temperature (25 °C). The microreactor was then heated to 300 °C and kept for 2 h. When the temperature decreased to room temperature, a heating programme was started where an Ar/O_2_ mixture (80%/20%) was introduced at a flow rate of 50 mL/min into the DMMP generation bottle, which was kept at 10 °C. While maintaining the continuous flow of Ar/O_2_/DMMP mixture into the microreactor, the temperature was increased from room temperature to 400 °C at a rate of 10 °C/min. The exhaust gases were qualitatively analysed online by using the mass spectrometer (HPR-20).

## 3. Results

### 3.1. Crystalline Structures

**Figure 2:**
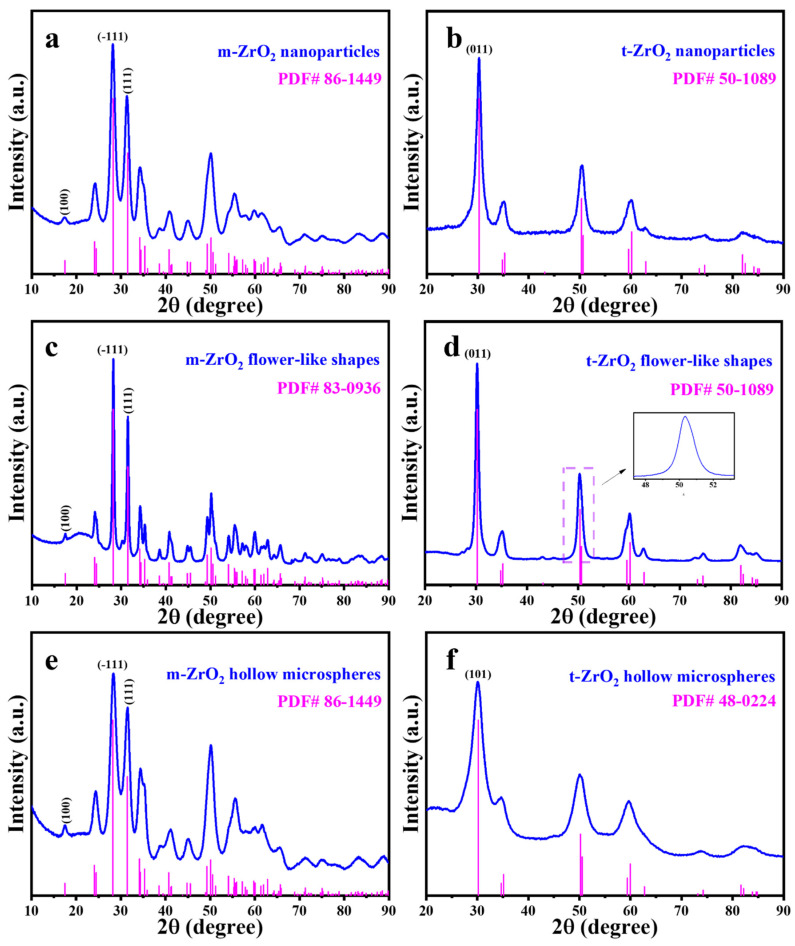
XRD spectra of ZrO_2_ catalysts: m-ZrO_2_ (**a**) nanoparticles, (**c**) flower-like shapes and (**e**) hollow microspheres; t-ZrO_2_ (**b**) nanoparticles, (**d**) flower-like shapes and (**f**) hollow microspheres.

**Figure 3:**
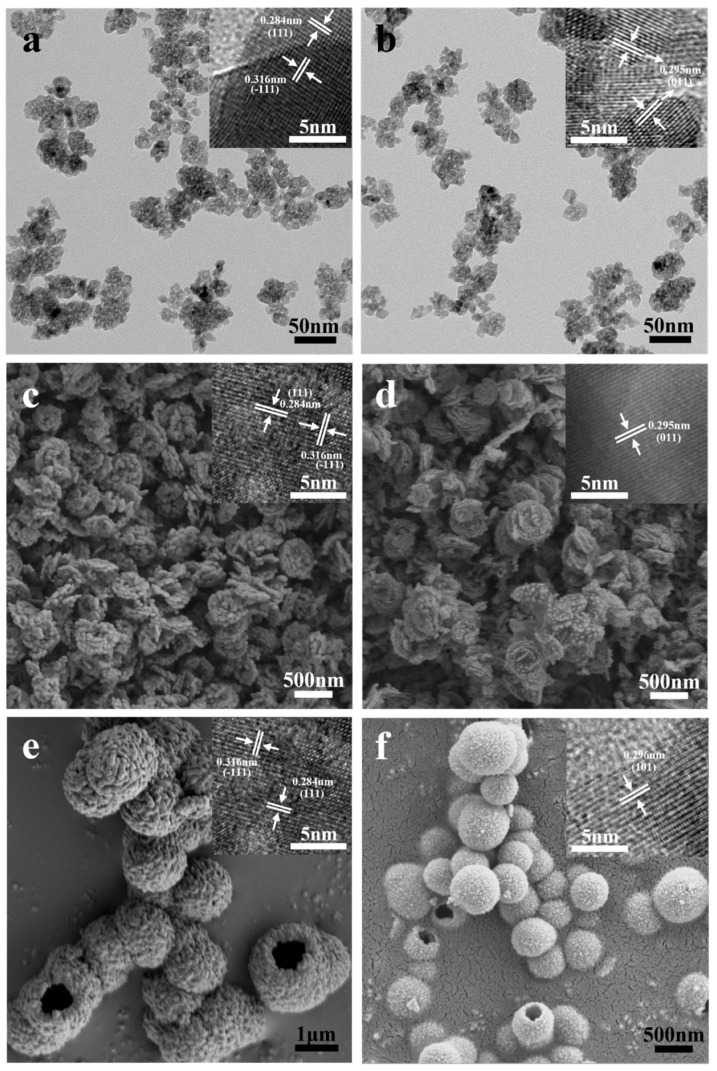
TEM and HRTEM images of ZrO_2_ catalysts: (**a**) m-ZrO_2_ nanoparticles and (**b**) t-ZrO_2_ nanoparticles; SEM and HRTEM images of ZrO_2_ catalysts: (**c**) m-ZrO_2_ with flower-like shapes, (**d**) t-ZrO_2_ with flower-like shapes, (**e**) m-ZrO_2_ with hollow microspheres, and (**f**) t-ZrO_2_ with hollow microspheres.

Figure 2 presents the XRD patterns of the six prepared catalysts, and Figure 3 shows the HRTEM images. The characteristic peaks of the m-ZrO_2_ nanoparticles (Figure 2a) and hollow microspheres (Figure 2e) showed good agreement with the XRD spectrum of JCPDS No. 86-1449. The HRTEM images of the m-ZrO_2_ nanoparticles (Figure 3a) and hollow microspheres (Figure 3e) showed clear lattice stripes with spacings of 0.316 and 0.284 nm corresponding to the (−111) and (111) crystal planes, respectively. The characteristic peaks of the m-ZrO_2_ flower-like shapes (Figure 2c) showed good agreement with the XRD spectrum of JCPDS No. 83-0936. The lattice stripes had spacings of 0.316 and 0.284 nm, corresponding to the (−111) and (111) crystal planes, respectively. The characteristic peaks of the t-ZrO_2_ nanoparticles (Figure 2b) and flower-like shapes (Figure 2d) showed good agreement with the XRD spectrum of JCPDS No. 50-1089. The t-ZrO_2_ flower-like shapes had an asymmetric peak near 2θ = 50°, which is consistent with the results of Shu et al. [23] and which confirms that t-ZrO_2_ was synthesised. The HRTEM images of the t-ZrO_2_ nanoparticles (Figure 3b) and flower-like shapes (Figure 3d) showed a lattice spacing of 0.295 nm corresponding to the (011) crystal plane. Meanwhile, the characteristic peaks of the t-ZrO_2_ hollow microspheres (Figure 2f) showed good agreement with the XRD spectrum of JCPDS No. 48-0224, and the HRTEM image (Figure 3f) showed a lattice spacing of 0.296 nm, which corresponds to the (101) crystal plane. These results indicate that pure m- and t-ZrO_2_ were successfully synthesised.

Figure 3 also shows the SEM and TEM images of the six prepared catalysts. The particle size distribution of these six ZrO_2_ catalysts has been calculated using Nano Measure software and fitted with the Gaussian function (Figures S1–S6). The m- and t-ZrO_2_ nanoparticles (Figure 3a,b, respectively) had similar particle sizes of approximately 5 nm (Figures S1 and S2). The m- and t-ZrO_2_ flower-like shapes (Figure 3c,d, respectively) had a layered structure where each layer comprised rough nanoparticles. Each layer had the size of approximately 350 nm (Figures S3 and S4) and thickness of approximately 50 nm. The m- and t-ZrO_2_ hollow microspheres (Figure 3e,f, respectively) were formed by the aggregation of small nanoparticles. Moreover, the m-ZrO_2_ hollow microspheres were of various sizes with a range of 0.5–2.5 µm (Figure S5), while the t-ZrO_2_ hollow microspheres had a uniform size distribution of 200–800 nm (Figure S5). While the hollow microspheres were often individual, and some were twinned, so these catalysts often had a rough and notched surface.

### 3.2. Catalytic Performance

**Figure 4:**
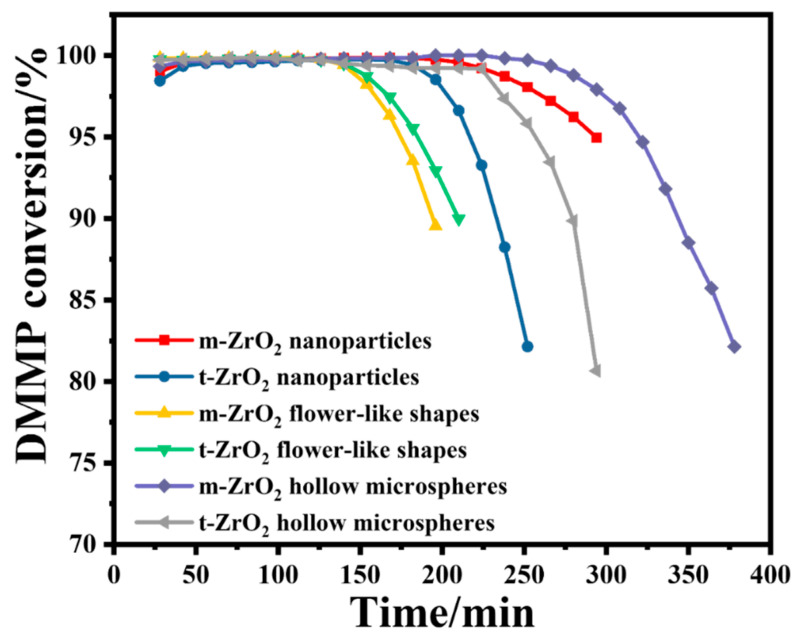
DMMP conversion rates over time of the six ZrO_2_ catalysts.

Figure 4 shows the DMMP conversion rates of the six prepared catalysts over time, and Table 1 summarises the corresponding protection times. In terms of morphology, the ZrO_2_ hollow microspheres had the longest protection time, followed by the ZrO_2_ nanoparticles and ZrO_2_ flower-like shapes. In terms of the crystalline phase, the m-ZrO_2_ nanoparticles and hollow microspheres exhibited longer protection times than their t-ZrO_2_ counterparts. In contrast, the m-ZrO_2_ and t-ZrO_2_ flower-like shapes had the same protection time. Table 2 provides a comparison of protection time on various catalysts reported in the literature.

In the initial stage of thermocatalytic decomposition, DMMP micro-penetration appeared, and the activity of the ZrO_2_ catalysts gradually increased to a normal level. This phenomenon is common in fixed-bed reactors, which can be attributed to a slight deactivation of ZrO_2_ during storage. However, ZrO_2_ can be reactivated when the catalytic temperature increases to a certain threshold. As the reaction progressed, when the catalytic time exceeded the maximum protection time, DMMP began to break through, and the DMMP conversion rate sharply decreased, which indicates that the catalyst rapidly deactivated. Table 1 lists the specific surface areas of the prepared catalysts. The protection times of the catalysts appear to be associated with their specific surface areas. This may be because catalysts with a larger specific surface area have a stronger DMMP adsorption capacity, which promotes the catalytic reaction and increases the protection time. Among the six catalysts, the m-ZrO_2_ hollow microspheres had the largest specific surface area by far of 100.1 m^2^/g, and they exhibited the best catalytic performance with a protection time of up to 266 min. The t- and m-ZrO_2_ flower-like shapes, m-ZrO_2_ nanoparticles and t-ZrO_2_ hollow microspheres had similar specific surface areas and the same protection time.

To avoid the effect of different catalyst loadings on the protection time, the mass specific treatment capacity (MSTC) was calculated based on parameters such as the catalyst filling quality, protection time, gas hourly space velocity and occurrence concentration. The m-ZrO_2_ hollow microspheres had the best MSTC (0.189 g_DMMP_/g_cat_), followed by the t-ZrO_2_ hollow microspheres (0.158 g_DMMP_/g_cat_), m-ZrO_2_ nanoparticles (0.145 g_DMMP_/g_cat_), and t-ZrO_2_ nanoparticles (0.121 g_DMMP_/g_cat_). The m-ZrO_2_ flower-like shapes (0.085 g_DMMP_/g_cat_) and t-ZrO_2_ flower-like shapes (0.087 g_DMMP_/g_cat_) had the worst MSTC, which is consistent with the results for the protection time. The surface area-specific treatment capacity (SSTC) was also calculated, and the t-ZrO_2_ flower-like shapes had the best SSTC, which can be attributed to it having the lowest specific surface area.

### 3.3. H_2_-TPR Analysis

**Figure 5:**
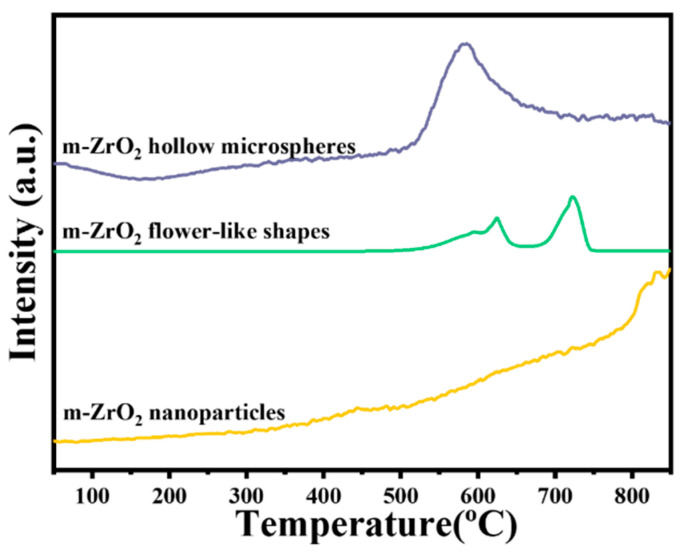
H_2_-TPR curves of the m-ZrO_2_ catalysts with different morphologies.

Overall, the m-ZrO_2_ catalysts had longer protection times than the t-ZrO_2_ catalysts. Thus, the surface chemical properties of the m-ZrO_2_ catalysts were further explored. Figure 5 shows the H_2_-TPR curves of the m-ZrO_2_ hollow microspheres, flower-like shapes and nanoparticles. The m-ZrO_2_ hollow microspheres exhibited a weaker reduction peak between 200 °C and 500 °C than the other morphologies, indicating that the m-ZrO_2_ hollow microspheres had relatively high catalytic oxidation activity at low temperatures. Moreover, the m-ZrO_2_ hollow microspheres had a strong reduction peak around 590 °C, whereas the m-ZrO_2_ flower-like shapes displayed three consecutive peaks between 520 and 740 °C. The m-ZrO_2_ nanoparticles not only exhibited a very weak characteristic peak around 450 °C but also exhibited a weak reduction peak between 500 and 750 °C. The hydrogen consumption (i.e., reduction peak area) of m-ZrO_2_ hollow microspheres was markedly greater than that of m-ZrO_2_ flower-like shapes and nanoparticles, which indicates that the m-ZrO_2_ hollow microspheres had stronger oxidation activity.

### 3.4. Thermocatalytic Decomposition Mechanism

**Figure 6:**
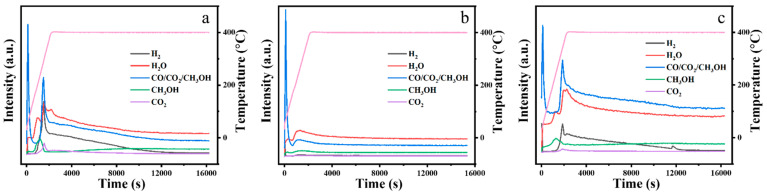
Mass spectrometry results for the exhaust gases from the thermocatalytic decomposition of DMMP by the m-ZrO_2_ catalysts: (**a**) nanoparticles, (**b**) flower-like shapes, and (**c**) hollow microspheres (The pink line is the curve of the heating program).

Figure 6 shows the mass spectrometry results for the exhaust gases of the m-ZrO_2_ catalysts. The three m-ZrO_2_ catalysts had the same gaseous products, including methanol, H_2_O, H_2_, and CO_2_. The formation of methanol is associated with the elimination of the methoxy group in DMMP, and methanol can further be oxidised to generate H_2_O, H_2_, and CO_2_ [29,30,31,32,33,34].

**Figure 7:**
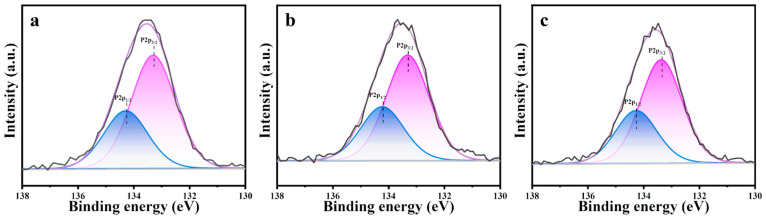
XPS spectra (P2p) of m-ZrO_2_ (**a**) nanoparticles, (**b**) flower-like shapes, and (**c**) hollow microspheres.

**Figure 8:**
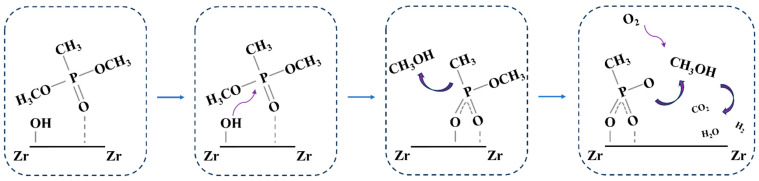
Proposed reaction mechanism for DMMP decomposition.

Figure 7 shows the X-ray photoelectron spectroscopy results for the m-ZrO_2_ catalysts, which were obtained to investigate the residues produced on the catalysts after the thermocatalytic decomposition. The P2p spectrum was detected on the surface of all three m-ZrO_2_ catalysts, which indicates that P-containing by-products remained on the catalysts after they were used. The P2p spectra were fitted to two peaks at 133.2 and 134.3 eV. The latter peak was attributed to the residual DMMP molecules on the m-ZrO_2_ surface after the thermocatalytic decomposition [35], and the former was ascribed to phosphorus oxide species remaining on the catalyst surface from the complete or incomplete decomposition of DMMP [35]. Based on results in the literature [33,34,36] as well as the exhaust gas and surface products after thermocatalytic decomposition, the reaction mechanism can be deduced as follows. DMMP molecules first adsorbed on the catalyst surface through the P=O bond, and the P–O bond in the P–OCH_3_ group is broken to generate gaseous methanol and solid phosphorus oxide by-products. The methanol is further oxidised to gaseous CO_2_, H_2_, and H_2_O, whereas the solid phosphorus oxide by-products are deposited on the catalyst surface, which leads to catalyst deactivation, as shown in Figure 8. 

## 4. Discussion

In this study, six types of ZrO_2_ catalysts with nanoparticle, flower-like shape and hollow microsphere morphologies in monoclinic and tetragonal phases were synthesized, and their thermocatalytic decomposition performance for DMMP was studied. In terms of protection time, m-ZrO_2_ catalysts exhibited superior performance to t-ZrO_2_ catalysts in the thermocatalytic decomposition of DMMP. For a given crystalline phase, ZrO_2_ hollow microspheres performed better than ZrO_2_ flower-like shapes and nanoparticles. Among the six catalyst materials, m-ZrO_2_ hollow microspheres exhibited the best MSTC and the longest protection time of 266 min at 400 °C, but t-ZrO_2_ flower-like shapes exhibited the best SSTC. The exhaust gases and surface by-products of the catalysts were analysed to deduce the deactivation mechanism, it is inferred that the reaction paths of the three morphologies of m-ZrO_2_ on the catalytic decomposition of DMMP are similar. The deposition of phosphorus oxide by-products on the catalyst surface led to the loss of catalyst active sites. The results of this study helped us to obtain a deep and systematic understanding of the thermocatalytic decomposition of DMMP by ZrO_2_ catalysts with different morphologies and crystalline phases. The findings are expected to provide guidance for designing high-performance ZrO_2_-based composite catalysts for the degradation of chemical warfare agents.

## Figures and Tables

**Table 1:** Specific surface area, filling quality, and catalytic performance of the six ZrO_2_ catalysts.

Morphology	Crystalline Phase	BET (m^2^/g)	Filling Quality (g)	Protection Time (min)	MSTC g_DMMP_/g_cat_	SSTC g_DMMP_/S_cat_
Nanoparticle	Monoclinic	88.7	0.4105	224	0.145	0.00067
	Tetragonal	66.1	0.4005	182	0.121	0.00073
Flower-like shape	Monoclinic	38.7	0.4392	140	0.085	0.00096
	Tetragonal	23.1	0.4265	140	0.087	0.00161
Hollow microsphere	Monoclinic	100.1	0.3753	266	0.189	0.00071
	Tetragonal	88.3	0.3762	224	0.158	0.00067

**Table 2:** A comparison of protection time on various catalysts.

Reference	Catalyst	Reaction Condition	Protection Time
Lee et al. [27]	Cu2-HA	400 °C, DMMP concentration 3.58 g/m^3^, flow rate 100 mL/min	7.5 h
	1.6%Pt-TiO_2_	300 °C, DMMP concentration 3.58 g/m^3^, flow rate 100 mL/min	8 h
Graven et al. [28]	0.5%Pt-Al_2_O_3_	299 °C, DMMP concentration 3.5 g/m^3^, flow rate 8.85 L/min	8 h
Cao et al. [26]	10% V/Al_2_O_3_	400 °C, DMMP concentration 1300 ppm, flow rate 50 mL/min	12.5 h
	1% Pt/Al_2_O_3_		8.5 h
	10% Cu/Al_2_O_3_		7.5 h
	Al_2_O_3_		4.0 h
	10% Fe/Al_2_O_3_		3.5 h
	10% Ni/Al_2_O_3_		1.5 h
	10% V/SiO_2_		25 h
Gao et al. [12]	CuO/γ-Al_2_O_3_	350 °C, DMMP concentration 4.0 g/m^3^, flow rate 100 mL/min	1.8 h
	CuO-1% CeO_2_/γ-Al_2_O_3_		2.1 h
	CuO-5% CeO_2_/γ-Al_2_O_3_		3.9 h
	CuO-10% CeO_2_/γ-Al_2_O_3_		1.8 h
Kong et al. [29]	2MCeO_2_np	300 °C, DMMP concentration 5.32 g/m^3^, flow rate 50 mL/min	5.8 h
	6MCeO_2_nr		7.0 h
	12MCeO_2_nr		8.1 h
	6MCeO_2_nc		3.5 h
	12MCeO_2_nc		6.3 h
Kong et al. [30]	CeO_2_	400 °C, DMMP concentration 8.46 g/m^3^, flow rate 100 mL/min	2.33 h
	10% Cu/Ce		4.2 h
	20% Cu/Ce		4.43 h
	50% Cu/Ce		5.36 h
	80% Cu/Ce		2.33 h
	CuO		0.93 h
This work	m-ZrO_2_ nanoparticles	400 °C, DMMP concentration 5.3 g/m^3^, flow rate 50 mL/min	3.73 h
	t-ZrO_2_ nanoparticles		3.03 h
	m-ZrO_2_ flower-like shapes		2.33 h
	t-ZrO_2_ flower-like shapes		2.33 h
	m-ZrO_2_ hollow microspheres		4.43 h
	t-ZrO_2_ hollow microspheres		3.73 h

## Data Availability

Data are contained within the article and Supplementary Materials.

## Supplementary Materials

The following supporting information can be downloaded at: https://www.mdpi.com/article/10.3390/nano14070611/s1, Figure S1. TEM image (a) and particle size distribution of m-ZrO_2_ nanoparticles (b). Figure S2. TEM image (a) and particle size distribution of t-ZrO_2_ nanoparticles (b). Figure S3. TEM image (a) and particle size distribution of m-ZrO_2_ with flower-like shapes (b). Figure S4. TEM image (a) and particle size distribution of t-ZrO_2_ with flower-like shapes (b). Figure S5. TEM image (a) and particle size distribution of m-ZrO_2_ with hollow microspheres (b). Figure S6. TEM image (a) and particle size distribution of t-ZrO_2_ with hollow microspheres (b).
