# Supplementary material for: Effects of the ZrO2 Crystalline Phase and Morphology on the Thermocatalytic Decomposition of Dimethyl Methylphosphonate
Source: Nanomaterials (Basel). 2024 Mar 30;14(7):611. doi: 10.3390/nano14070611 (PMC11013148; doi:10.3390/nano14070611)
Supplement: Supplementary file 1 [file nanomaterials-14-00611-s001.zip › nanomaterials-2889202-supplementary.pdf]

Supporting Information

Effects of the ZrO_2 Crystalline Phase and Morphology on the Thermocatalytic Decomposition of Dimethyl Methylphosphonate

Xuwei Wang [1,2], Peng Sun [1,2], Ziwang Zhao [2], Yimeng Liu [2], Shuyuan Zhou [2,*], Piaoping Yang [1,*] and Yanchun Dong [2,*]

[1] Key Laboratory of Superlight Materials and Surface Technology, Ministry of Education, College of Material Sciences and Chemical Engineering, Harbin Engineering University, Harbin 150001, China; wangxw5710@hrbeu.edu.cn (X.W.); pengs@hrbeu.edu.cn (P.S.)

[2] State Key Laboratory of NBC Protection for Civilian, Beijing 102205, China; zhaoziwang1123@163.com (Z.Z.); 202311087233@mail.scut.edu.cn (Y.L.)

* Correspondence: zhoushuyuan@sklnbcpc.cn (S.Z.); yangpiaoping@hrbeu.edu.cn (P.Y.); dongyanchun@sklnbcpc.cn (Y.D.)

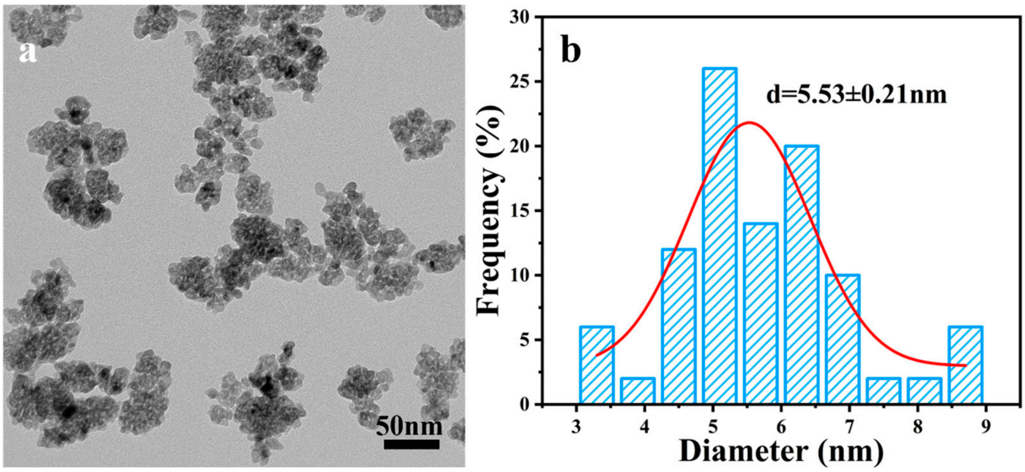

Figure S1. TEM image (a) and particle size distribution of m-ZrO_2 nanoparticles (b).

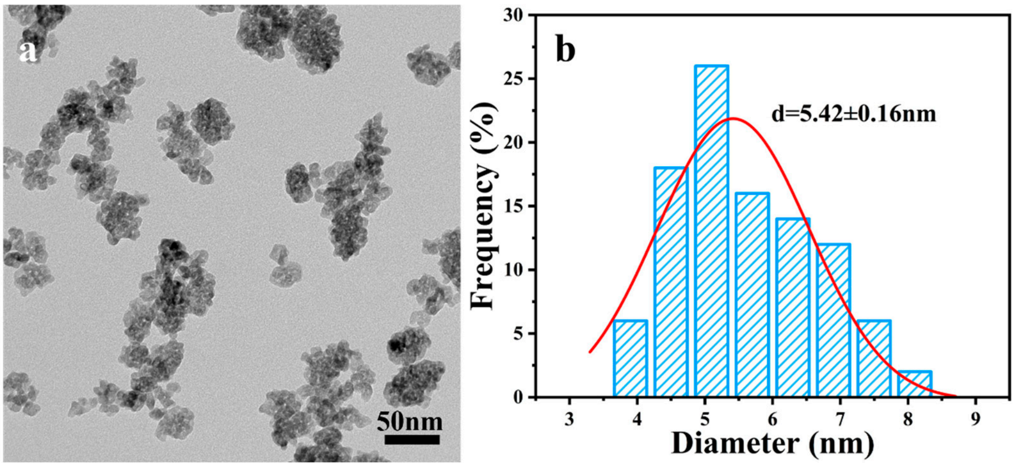

Figure S2. TEM image (a) and particle size distribution of t-ZrO_2 nanoparticles (b).

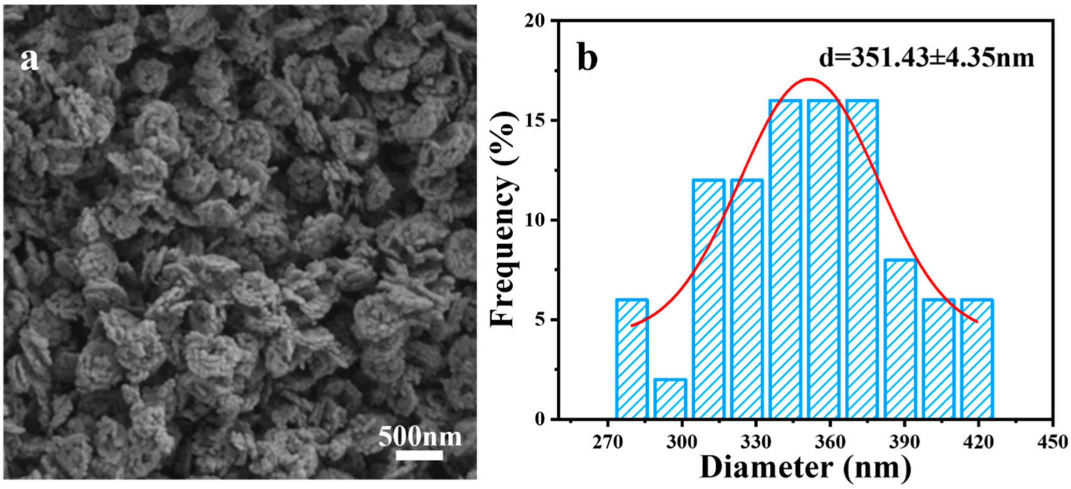

Figure S3. TEM image (a) and particle size distribution of m-ZrO_2 with flower-like shapes (b).

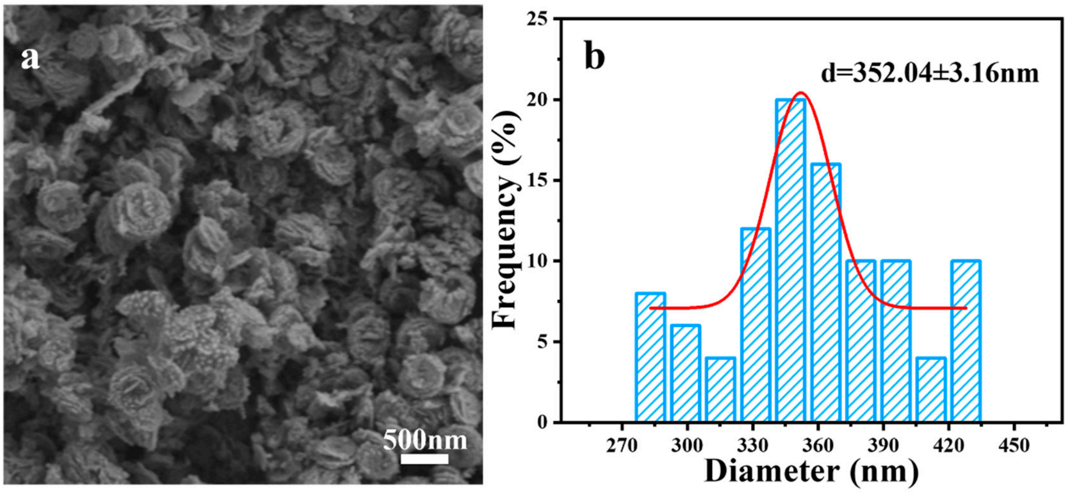

Figure S4. TEM image (a) and particle size distribution of t-ZrO_2 with flower-like shapes (b).

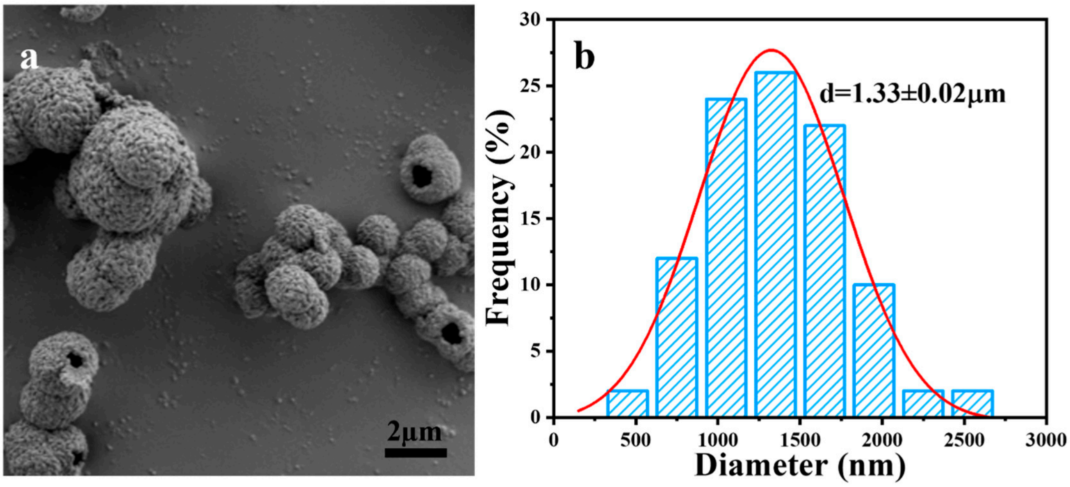

Figure S5. TEM image (a) and particle size distribution of m-ZrO_2 with hollow microspheres (b).

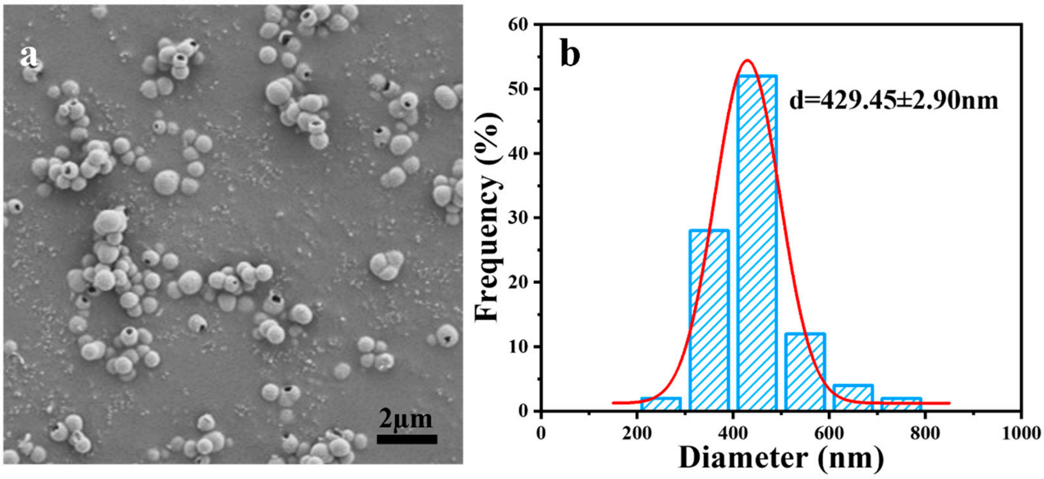

Figure S6. TEM image (a) and particle size distribution of t-ZrO_2 with hollow microspheres (b).
